# Predicting and Promoting Human Bone Marrow MSC Chondrogenesis by Way of TGFβ Receptor Profiles: Toward Personalized Medicine

**DOI:** 10.3389/fbioe.2020.00618

**Published:** 2020-06-26

**Authors:** René Rothweiler, Valentina Basoli, Fabian Duttenhoefer, David Kubosch, Rainer Schmelzeisen, Brian Johnstone, Mauro Alini, Martin James Stoddart

**Affiliations:** ^1^Regenerative Orthopaedics, AO Research Institute Davos, Davos, Switzerland; ^2^Department of Oral and Maxillofacial Surgery, Faculty of Medicine, Medical Center - University of Freiburg, University of Freiburg, Freiburg im Breisgau, Germany; ^3^Department of Orthopedics and Trauma Surgery, Faculty of Medicine, Medical Center - University of Freiburg, University of Freiburg, Freiburg im Breisgau, Germany; ^4^Department of Orthopaedics and Rehabilitation, Oregon Health & Science University, Portland, OR, United States

**Keywords:** mesenchymal stem cell, TGF receptor, chondrogenic differentiation, receptor ratio, personalized medicine

## Abstract

The use of human mesenchymal stromal cells (hMSCs) for cartilage regeneration has been hampered by the inherent donor variation of primary monolayer expanded cells. Although CD markers are typically used to characterize cell populations, there is no correlation between CD marker profile and functional outcomes. Therefore, we aimed to discover novel predictive MSC chondrogenesis markers. The chondrogenic potential of primary human bone marrow MSCs (hBMSCs) over multiple passages was assessed by standard pellet culture. We confirmed that the ratio of TGFβ-RI/TGFβ-RII at the time of cell recovery from the tissue culture plastic reliably predicted chondrogenic potential. Furthermore, it is possible to prospectively characterize any human BMSC cell population as responders or non-responders with respect to chondrogenic differentiation potential. Transient increase of the ratio with siRNA knockdown of TGFβ-RII reproducibly recovered the chondrogenic differentiation ability of non-responsive MSCs. Together this offers an opportunity to produce a more functionally characterized cell population for use in autologous cartilage repair therapies.

## Introduction

Despite the promise of human bone marrow derived stromal cells (hBMSCs) in the field of regenerative medicine, assays predictive of cell function have remained elusive. Several studies show the *in vitro* potential of MSCs to differentiate into chondrocytes under specific stimulations (Wakitani et al., 1994; Cassiede et al., 1996; Yoo et al., 1998); however, the chondrogenic commitment of human MSCs is highly variable. While it has been established that factors such as *in vivo* age and *in vitro* aging, in the case of monolayer expanded/ selected cells, play roles in the variability, there remains much to be gleaned concerning the differences between MSC populations from different individuals. The consequence of our current incomplete picture of the causes of MSC variability is that to date, it is not possible to predict the outcome of chondrogenic differentiation of a specific donor. This is a clinical challenge as cell based therapies are expensive to administer and without adequate patient stratification they become financially unviable. The expression levels of certain cell surface markers (e.g., Stro-1, CD73, CD105, or CD90) have been associated with MSCs (Stewart et al., 2003; Battula et al., 2009) but none are known to be predictive of stemmness or commitment and do not correlate with the final yield and quality of chondrogenic differentiation [e.g., (Cleary et al., 2015)]. Furthermore, most marker profiles are similar for all cells of mesenchymal origin (Whitney et al., 2009) but hMSCs from different origins have been shown to retain epigenetic memory and display functional differences *in vivo*. These differences are not reflected by the marker profile *in vitro* (Sacchetti et al., 2016). For this reason new methods for predicting the functional potential of hMSCs are urgently needed (McLeod and Mauck, 2017).

During chondrogenic commitment *in vitro*, TGFβ is one of the key factors involved in the determination of cell fate. MSCs express TGFβ receptor type II (TGFβ-RII) on their membrane surfaces, which recognizes and binds TGFβ. This activated complex recruits a type I receptor dimer (TGFβ-RI) creating a phosphorylated hetero-tetrameric complex that progressively activates signaling pathways via SMAD proteins (Grimaud et al., 2002). SMADs translocate into the nucleus and promote gene regulation (Shi and Massague, 2003) through modulation of transcriptional co-activators and co-repressors in a cell type specific manner (Nakao et al., 1997; Massague, 2012). Canonical TGFβ-RI activation is known to mediate the signal via intracellular Smad 2/3; whereas ACVRL-I, also known as ALK1 - an alternative receptor regulated by TGFβ–promotes Smad 1/5/8 phosphorylation (Nakao et al., 1997; Chen and Massague, 1999), a pathway generally associated with hypertrophy.

It has been shown that TGFβ-RI expression and Smad2 phosphorylation decrease dramatically in aged murine cartilage (Blaney Davidson et al., 2005) with a concomitant increase in ACVRL-I expression, resulting in dysregulated chondrogenesis caused by an alteration in TGFβ downstream signaling (Blaney Davidson et al., 2009). Acting on this observation, we investigated the expression of numerous TGFβ receptors in MSCs from a cohort of human donors at different stages of *in vitro* expansion. The aim was to determine a marker profile that reliably predicts the chondrogenic potential of hMSC populations. Based on initial data we then examined in more detail the TGFβR1/TGFβR2 ratio profile in additional donors as a possible predictable indicator of quality and yield. To demonstrate a functional role and influence of different TGFβ receptors involved in the chondrogenic fate, we modulated the ratio in order to improve the chondrogenesis in MSCs that showed a limited chondrogenic potential.

## Materials and Methods

### Human Mesenchymal Stromal Cell Isolation From Fresh Bone Marrow

Bone marrow from 20 different anonymous human donors (range min 18 years, max 85 years, Average 57.71 ± 19.34 years) was harvested after informed consent (Ethical approval: Freiburg, EK-326/08). A known co-morbidity was considered an exclusion criterion. Fresh bone marrow was diluted 1:4 and layered on top of Ficoll, in a proportion of 2.6 ml of Ficoll per ml of undiluted marrow. After centrifugation at 500 g for 20 min, the mononuclear cell-containing interface was recovered, and cells were counted using the Cell Scepter 2.0 Automated Cell Counter (Millipore). Isolated cells were seeded at a density of 50,000 cells/cm^2^ into 300 cm^2^ tissue culture flasks in Minimum Essential Medium Eagle, Alpha Modification (α-MEM; Gibco, UK) containing 10% fetal bovine serum (Sera Plus, PAN-Biotec 3702-P12812 Aidenbach, Germany), 100 U/mL penicillin, and 100 μg/mL streptomycin (Gibco, UK), and 5 ng/ml recombinant human basic fibroblast growth factor (bFGF, Fitzgerald Industries International, Acton, MA, USA). Cells were maintained at 37°C in 5% CO_2_, 85% humidity atmosphere. Medium was refreshed every 2nd day. After 4 days, non-adherent hematopoietic cells were removed to select the mesenchymal stromal cell (hMSC) population.

### Passaging

hMSCs were cultured from passage 0 up to passage 10, with an initial seeding density of 3,000 cells/cm^2^ in 300 cm^2^ tissue culture flasks, in the conditions described above. Upon reaching 80% confluency, images of cells were taken in order to record their morphology. Cultures were passaged using Trypsin-EDTA (0.5%) (ThermoFisher, UK) for 5 min at 37°C. For the deactivation of trypsin 1:3 growth medium containing 10% fetal bovine serum was used, cells were then centrifuged at 400 G for 5 min. The resultant pellet was used for further expansion or for RNA isolation to evaluate the TGFβ receptor expression.

### Chondrogenic Differentiation

Chondrogenic differentiation of hMSCs was performed in 3D pellet culture. A quantity of 2 x 10^5^ hMSCs per pellet were seeded in V-bottom 96-well plates (Corning, Corning, NY, USA). To prevent possible cell adhesion on the bottom, the plate was pre-coated with 20 μl of 1% agarose. Cells were centrifuged for 5 min at 500 g in order to form the pellets. Chondrogenic differentiation medium contained DMEM high glucose (Gibco, UK), 1% non-essential amino acids (ThermoFisher, UK), 1% ITS+ (Corning, NY, USA), 100 nM dexamethasone (Sigma-Aldrich, Germany), 10 ng/ml TGF-β1 (Fitzgerald Industries International, Acton, MA, USA) and 50 μg/ml ascorbic acid-2 phosphate (Sigma-Aldrich, Germany). The control growth medium contained DMEM high glucose (Gibco, UK), 1% non-essential amino acids (ThermoFisher, UK), 1% ITS+ (Corning, NY, USA). The medium was replaced every second day and pellets were harvested for further analyses after 28 days.

### Transfection and Receptor Silencing

In order to demonstrate the role of TGFβ-Rs during chondrogenic commitment and their relevance during TGFβ signaling pathway activation, we transiently inhibited TGFβ-RI, TGFβ-RII, and ACVRL-I. According to manufactures' protocol using the NEON transfection system: hMSC were resuspended in Buffer R at a final concentration of 0.5 × 10^7^ cells/ml. Cells were transfected with either siTGFβ-RI (Ambion, cat#4427038), siTGFβ-RII (Ambion, cat#AM51331), siACVRL-I (Ambion, cat# 4427037) at 25 nM, or siNegative (scramble control) (Ambion, cat# 4390846) by electroporation using a 990 pulse voltage, 40 ms pulse width for one pulse number using a 100 μl pipette tip. Cells were then transferred into chondrogenic medium or control medium in absence of antibiotics.

### Real-Time Quantitative PCR Analysis

Total RNA was isolated from adherent hMSC cells after trypsinization during passaging and from 3D chondrogenic induced pellets at day 0 and 28 using TRI Reagent® Solution (Molecular Research Center MRC, cat. # TR-118) according to the manufacturer's protocol. RNA quantity was measured using a NanoDrop 1000 Spectrophotometer (Thermo Fisher, UK). For reverse transcription, TaqMan Reverse Transcription Kit (Applied Biosystems, Foster City, USA) was used. The RT reaction was carried out at 25°C for 10 min, followed by 1 h at 42°C and stopped by heating for 5 min at 85°C. qPCR reactions were set up in 10 μL reaction mixtures containing TaqMan Universal Master Mix (Thermo Fischer, UK), Primer and Probe or AssayOnDemand, DEPC-H20 and cDNA template. The reaction program was set up as follows: 50°C for 2 min, 95°C for 10 min, and 40 cycles of 95°C for 15 s followed by an annealing/extension step at 60°C for 1 min. qPCR analysis was performed using QuantStudio 6 Flex Real-Time PCR System (Life Technologies, Carlsbad, USA). Duplicates were used for each target gene (technical replicates) and triplicates for each donor (biological replicates).

The relative expression of TGFBR1, TGFBR2, BMPR1A, BMPR1B, BMPR2, ACVR1, ACVR1B, ACVR1C, ACVR2A, ACRV2B, ACVRL1 during expansion in monolayer was determined using the 2^(−Δ*ΔCT*)^ method, with ribosomal protein large, P0 (RPLP0) as reference gene and P2 RNA as the baseline.

The relative expression of RUNX2, SOX9, ACAN, MMP13, COL2A1, COL10A1 during chondrogenic differentiation was determined using the 2^(−ΔΔ*CT*)^ method, with ribosomal protein large, P0 (RPLP0) as reference gene and day 0 RNA as the baseline. Primer and probe sequences as well as Order Numbers of Assays-on-Demand (Applied Biosystems) are listed in the Supplemental Table 1.

To predict the chondrogenic function of an individual cell population the P2 baseline was omitted and the following calculation used.

The ratio of TGFβ-R expression was calculated using ΔCt values:

$$\begin{array}{l} R=2^{-\left(\Delta Ct_{1}-\Delta Ct_{2}\right)} \end{array}$$

Where Δ*Ct*_1_ = Ct hTGFβ-RI – Ct hRPLP0 and Δ*Ct*_2_ = Ct hTGFβ-RII—Ct hRPLP0.

### Histological Staining

At day 28, samples were fixed in 70% methanol. Cryosections were cut with a thickness of 8–10 μm. For Safranin-O staining, samples were first stained with Weigert's Haematoxylin for 10 min, followed by a 6 min stain with Fast Green and a 15 min stain with Safranin-O. After dehydration with increasing concentrations of ethanol, samples were coverslipped with the use of xylene.

For collagen II staining a monoclonal antibody (CIICI, DSHB, Iowa, USA) was used. After incubating slides in methanol for 30 min, nonspecific binding sites were blocked with horse serum (Vector Laboratories #S-2000; Dilution 1:20) for 1 h. Primary antibody was then added for 30 min (dilution 1:6) followed by an incubation in biotinylated anti-mouse IgG (H+L) secondary antibody (Vector Laboratories #BA-2001; dilution 1:200) and a second incubation in Vectastain Elite ABC Kit (Vector Laboratories #PK-6100). ImmPACT DAB solution (Vector Laboratories #SK-4105) was added as substrate for peroxidase for 4 min. Counterstaining was performed using Mayer's Haematoxylin (Fluka #51275) for 20 s. Samples were dehydration with increasing concentrations of ethanol and coverslipped. For the negative control, the respective samples were stained in parallel without addition of primary antibody.

The histological sections were observed using Zeiss AxioPlan 2 Microscope (Zeiss Microscopy GmbH, Jena, Germany) with objective 10X/0.50. and graded as previously published (Grogan et al., 2006). Pictures were acquired using RGB camera 1X (16 bit) and Axiocam Software (Zeiss Microscopy GmgH, Göttingen, Germany). Grading of the safranin O staining was ranked on a scale of 1–10 as assessed by four independent blinded evaluators according with Bern Score (Grogan et al., 2006) (Supplementary Table 2).

### Statistics

Statistical analysis was performed using GraphPad Prism 7.03 software. Non-parametric two-way ANOVA in conjunction with Tukey's multiple comparison test was applied. *P* < 0.05 was considered as statistically significant. A two-way ANOVA was used to evaluate distribution and homogeneity variance in the groups; the Tukey's multiple comparison was used to evaluate the means the different groups. For each experimental setup triplicates were done (biological triplicates). Analysis were done in duplicates (technical duplicates). A total number of 20 donors have been cultured from passage 2 to passage 10. Mean and standard deviation were calculated from the results. Spearman's rank correlation coefficient for the non-parametric measurement of the relationship between two rank variables (RATIO, Histological score, Chondrogenic Markers) has been described using a monotonic function using GraphPad Prism 7.03 software. Receiver operating characteristic curve (ROC curve), for the prediction study for each instance has been made based on a continuous random variable created by plotting the donors (based on TGFBR1/TGFBR2 ratio and histological score) responders against non-responders, in order to calculate the cut-off AUR (Arbitrary Unit Ratio). In our system we considered a binary classification, in which the outcomes are labeled either as responder or non-responder. We assumed that values with 100% sensitivity are associated (no false negatives/non-responders) to true good chondrogenesis and 100% specificity (no false positives/responders) are associated to poor chondrogenesis. ROC Curve was performed using GraphPad Prism 7.03 software.

## Results

### TGFβ-Receptor Expression Screening Over Passaging for Predictable Markers

**Figure 1:**
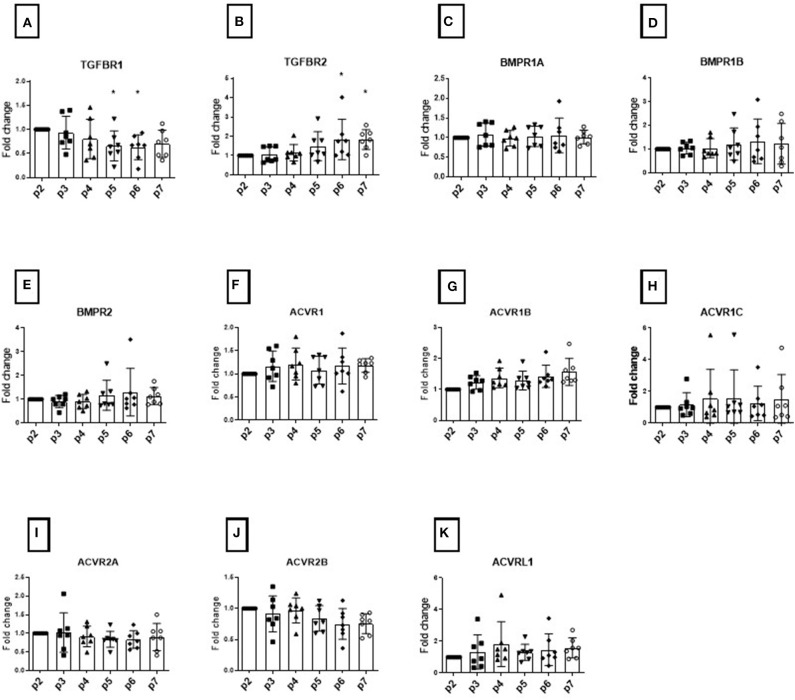
Modulation of TGFB receptors during monolayer expansion of hBMSCs from passage 2 to passage 7. Cells from different donors were cultured in conventional expansion medium. The amounts of TGFBR1 **(A)**, TGFBR2 **(B)**, BMPR1A **(C)**, BMPR1B **(D)**, BMPR2 **(E)**, ACVR1 **(F)**, ACVR2B **(G)**, ACVR1C **(H)**, ACVR2A **(I)**, ACVR2B **(J)**, ACVRL1 **(K)** mRNA was normalized to ribosomal protein large, P0 (RPLP0). The mRNA expression of cells cultured over the passaging were plotted as 2^(−ΔΔ*Ct*)^ (mean ± *SD*; *n* = 7) as a fold change to the BMSC in passage 2, the dots shows the different donors, *p < 0.05 compared to P2.

Initially using 7 donors, we focused on the study of TGFβ-R and BMP-R variation over passage within the donor cohort (considering all donors together) using the 2^(−ΔΔ*CT*)^ method and samples at passage 2 as calibrator (Figure 1). We compared the means and standard deviations up to passage 7 and investigated whether changes in the expression profiles of TGFβ-Rs and BMP-Rs during cell passaging could be associated with changing chondrogenic potential (Figure 1). As expected, there was a high standard deviation (*SD*) among the donors, and several receptors displayed an increase in *SD* with increasing passaging, leading us to investigate each receptor at the individual donor level.

The TGFβR1 (Figure 1A) and TGFβR2 (Figure 1B) expression levels changed during *in vitro* passaging. TGFβ-R1 expression levels tended to decrease over time, while TGFβ-R2 levels showed a concomitant increase in all donors analyzed, showing a large standard deviation. These changes however were not absolute with the opposite seen for some donors at some passages. These changes did not reach significance. We found that the mean expression BMPR1A, BMPR1B, and BMPR2 was unaltered over time among donors (Figures 1C–E). However, the *SD* of BMPR1B and BMPR2 was seen to increase over passages.

The serine/threonine-protein kinase receptors family (ACVR), another family belonging the TGFB superfamily, did not show any significant change in expression over time that would account for possible donor variability (Figures 1F–K). In order to assess changes with respect to function, histological analysis was performed at each passage. None of the changes in individual receptor profile correlated with the resulting chondrogenic differentiation (Figure 1).

**Figure 2:**
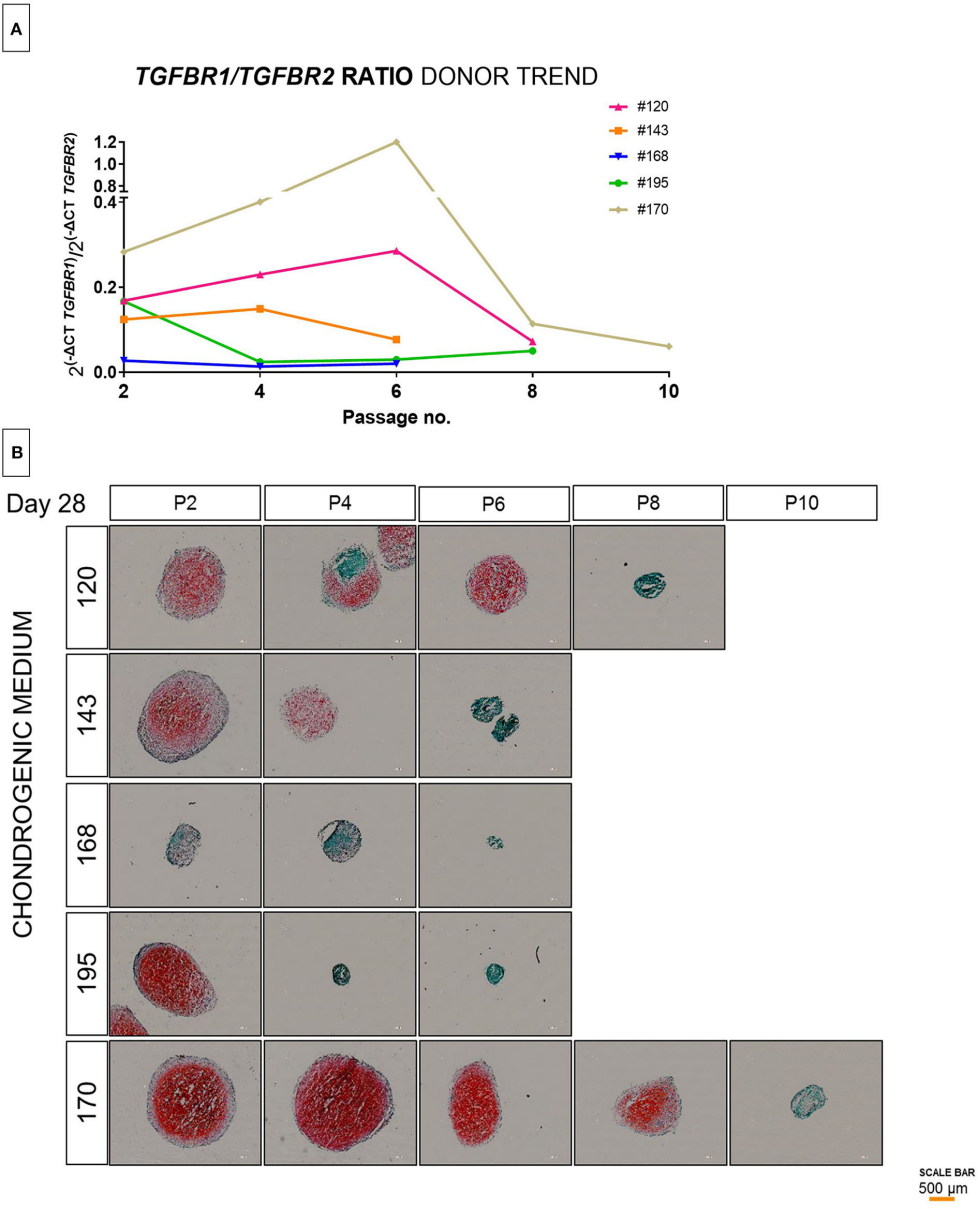
Evaluation of TGFBR ratio in extended population during monolayer expansion of hBMSCs from passage 2 to passage 10. Cells from different donors were cultured in conventional expansion medium. The amounts of TGFBR1/TGFBR2 ratio mRNA was normalized to ribosomal protein large, P0 (RPLP0). The mRNA expression of cells cultured over the passaging were plotted as 2^[−(Δ*Ct*_1−Δ*Ct*_2)]^ **(A)**, the lines shows the trend of individual donors over passaging **(B)**. Histological representation by safranin-O and fast green of 5 donors correlating to the initial observation of TGFBR1/TGFBR2 ratio and outcome of differentiation **(C)**. Red scale bar is 200 μm.

As TGF superfamily receptors heterodimerize, we then investigated whether ratios of expression correlated with chondrogenic differentiation. We observed the TGFBR1/TGFBR2 reliably correlated with the pellet culture histology on day 28 (Figure 2). The ratio at the time the cells were taken from the cell culture plastic correlated with the chondrogenic potential as assessed by safranin O staining. Additionally, we calculated the ratio without using P2 as a normalizer to allow a value to be generated from a single population of cells [$R=2^{-\left(\Delta Ct_{1}-\Delta Ct_{2}\right)}$]. The ratio was evaluated directly after cell harvest from tissue culture plastic. As expected, there was a trend toward a general decrease in ratio during *in vitro* aging, although for occasional populations a transient increase was observed. While the rate of change varied depending on the donor (Figure 2A), the ratio at the time of cell harvest correlated with the chondrogenic potential as assessed by safranin O staining (Figure 2B).

To validate these findings, we further assessed by molecular analysis of the TGFBR1/TGFBR2 ratio and the chondrogenic potential of additional donors from passage 2 to passage 10. Although the cells from different donors were isolated in the same way and maintained in the same conditions, it was evident that high donor variability is independent of passage number. The ratio between TGFBR1/TGFBR2 is highly donor dependent (Figure 2A), is not normally distributed over passaging and can be strongly affected from one passage to another (Figure 2B). Indeed, as is shown in Figure 2, the change in chondrogenic differentiation over time is donor dependent. Some donors considered good (e.g., #170) maintained a high yield of differentiation over time, while other donors considered bad (e.g., #168) already in the early phase showed a poor chondrogenesis (Figure 2). In addition, we observed, that some donors that show a high potential at lower passages (#195), drastically decrease the chondrogenic potential in a subsequent passage. This suggests, that although a donor may exhibit functional chondrogenesis at early passages, the change in chondrogenic potential over time cannot be reliably estimated.

**Figure 3:**
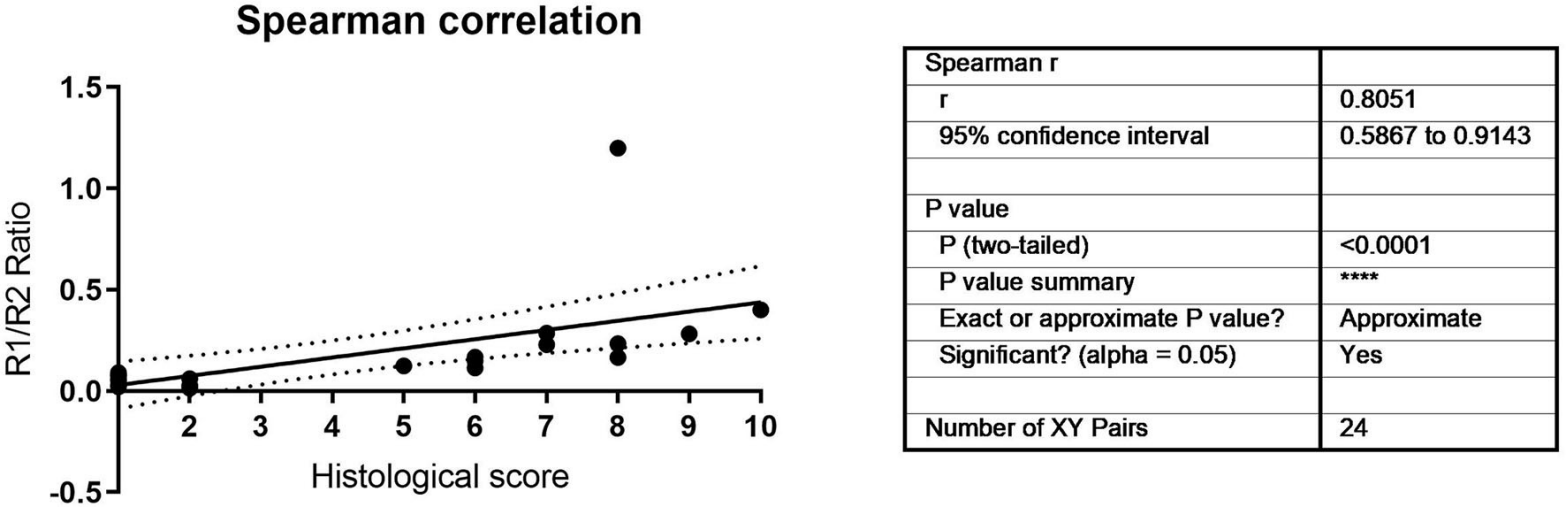
Histological and molecular Spearman correlation between TGFBR1/ TGFBR2 ratio and histological scores.

**Figure 4:**
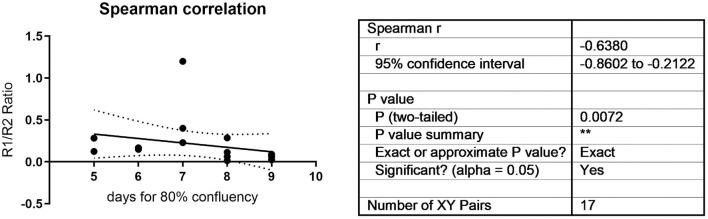
Time to 80% confluency was correlated to the TGFBR1/ TGFBR2 ratio. A statistically significant negative correlation was observed (r = −0.638, *P* = 0.0072).

To validate the correlation, a further 24 samples from multiple donors and multiple timepoints were analyzed, and the ratio correlated to histological outcomes ranked on a scale of 1–10 as assessed by four independent blinded evaluators according to the Bern Score (Grogan et al., 2006) (Figure 3, Supplementary Table 2). This score incorporates elements that consider the variability that can be seen within pellets, such as non-uniform staining. (Supplemental Table 2). The TGFBR1/ TGFBR2 ratio strongly correlated with the histological score (Figure 3, *r* = 0.8051, *P* < 0.001). It has been proposed that growth rate in monolayer correlates with MSC function. During monolayer expansion cells were harvested at 80% confluency. We used the number of days to achieve this mark as a proxy of cell doubling time, with more days required to 80% confluency being indicative of a slower growth. A small but statistically significant negative correlation between time to 80% confluence and ratio was detected, with slower growing cells performing worse (Figure 4). No correlation between donor age and chondrogenic potential was observed (data not shown).

### Identification of Cut-Off Value

**Figure 5:**
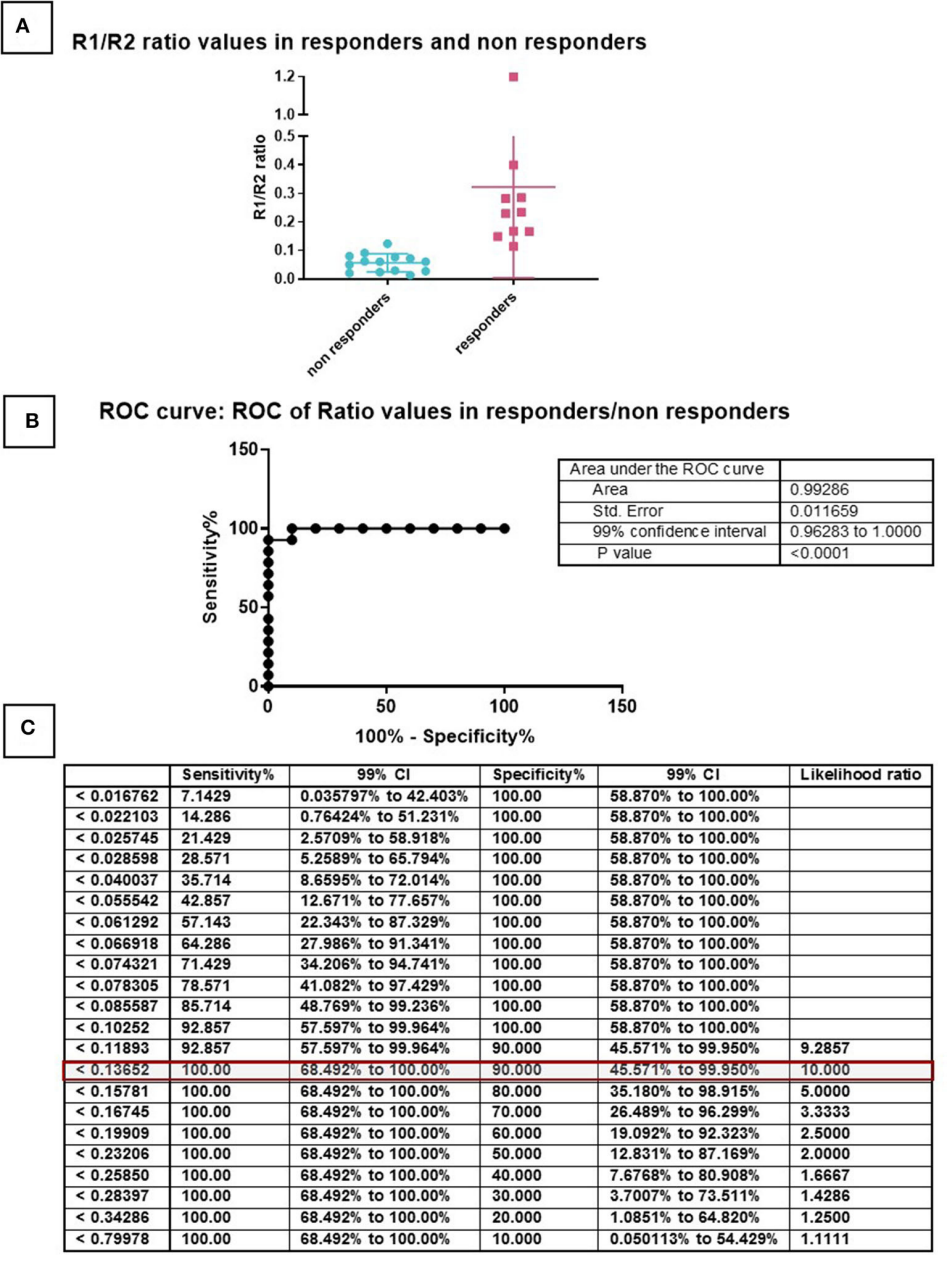
ROC curve for the determination of a cut-off. Samples were divided in two groups based on the histological score and TGFBR1/TGFBR2 ratio **(A)**. Non-responders have score 1–5 (green), Responders have score 6–10 (red). This division made it possible to generate a ROC curve **(B)** and calculate cut-offs for specificity and sensitivity **(C)**.

To identify the cut-off value below which the population is no longer chondrogenic, the 24 samples were then separated based on the histological evaluation, with histological scores between 1 and 5 considered poorly chondrogenic, while donors with a histological score between 6 and 10 were considered highly chondrogenic (Figure 5). We divided the two cohorts based on non-responders and responders for the respective TGFB ratio in order to evaluate a precise cutoff number and by ROC analysis the value of 0.136 was established (Figure 5C). This value correctly predicts 23 of the 24 samples (96%), with one sample being a false negative, having a ratio of 0.123991 but was still chondrogenic (Supplementary Table 3).

**Figure 6:**
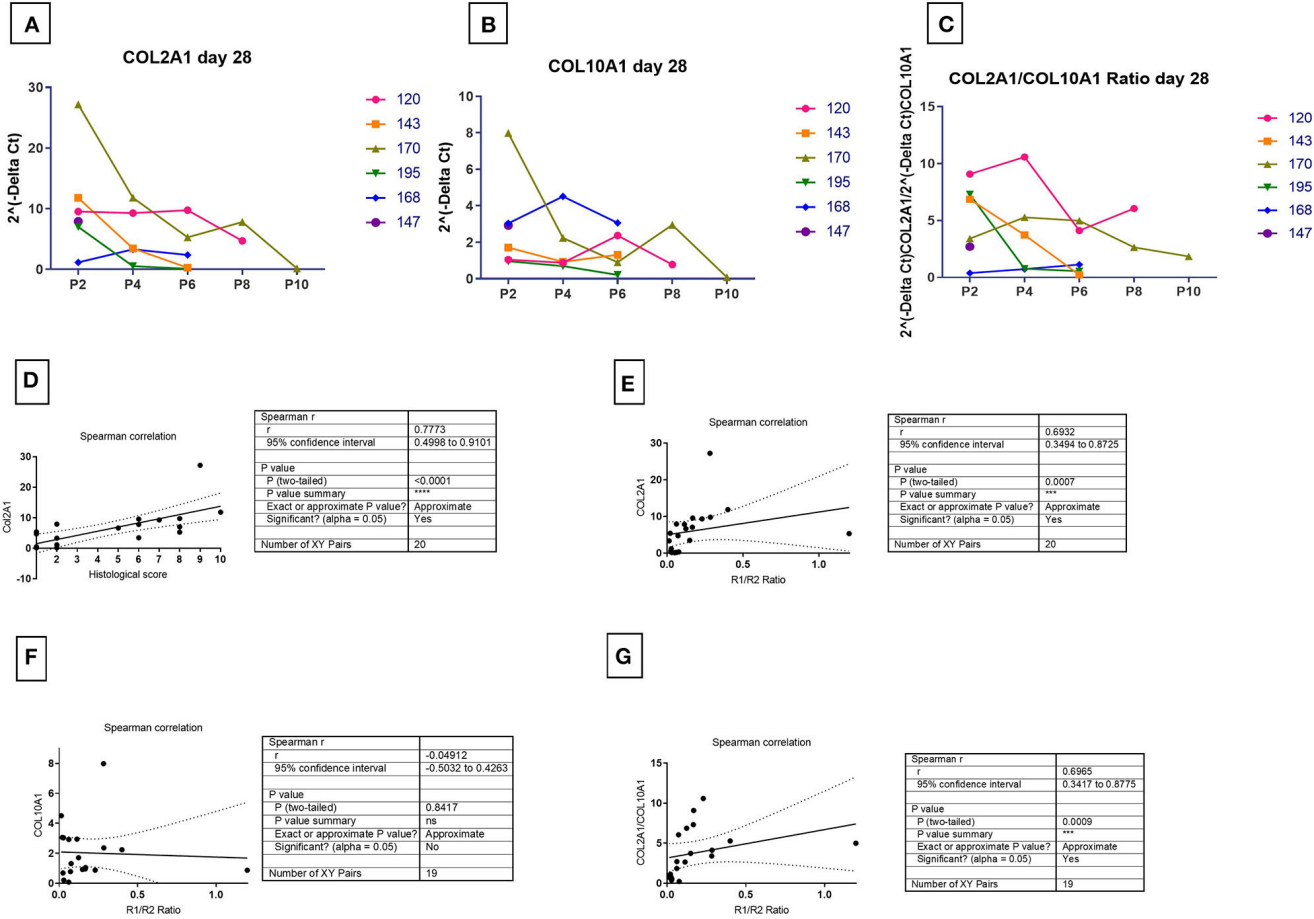
Correlation of marker receptors ratio and chondrogenic outcome markers. The COL2A1 **(A)**, COL10A1 **(B)**, COL2A1/COL10A1 ratio **(C)** for mRNA at 28 days in chondrogenic medium was normalized to ribosomal protein large, P0 (RPLP0). The mRNA expression of receptors over time were plotted as 2^(−Δ*Ct*)^. Spearman correlation for the detection of significance of hypothesis between R1/R2 and chondrogenic marker expression was done between the amount of COL2A1 and histological score **(D)**, the amount of COL2A1 and R1/R2 ratio **(E)**, the amount COL10A1 and R1/R2 ratio **(F)**, and COL2A1/COL10A1 ratio and R1/R2 ratio **(G)**. One sample had non-detectable levels of COL10A resulting in an *n* = 19 [Donor 195 P6- non-chondrogenic (see Supplementary Table 3)].

The expression of COL2A1 (Figure 6A), a chondrogenic marker associated with chondrogenesis also strongly correlated with the histological score (Figure 6D, *P* < 0.001) and the TGFBR1/ TGFBR2 ratio (Figure 6E, *P* = 0.002). In contrast, COL10A1 expression was not correlated to the TGFR1/TGFBR2 ratio (Figures 6B,F, *P* = 0.9488). That stated, the TGFR1/ TGFBR2 ratio strongly correlated with the COL2/COL10 ratio (Figures 6C,G, *P* = 0.004). Combined with the lack of correlation to COL10 expression, it suggests that increasing ratio is predictive of a more stable chondrogenesis.

### Recovery of Chondrogenic Phenotype

The use of AUR allows the prediction of hMSC chondrogenic outcome prior to the induction of differentiation. Of the 24 samples investigated in detail (Supplementary Table 3) 13 did not show any chondrogenic potential, and in each case, this could be predicted by the receptor ratio on the day of cell harvest. However, in order to test whether the receptor ratio was causally associated with the fate of hMSCs, three receptors (TGFBR2, TGFBR1, and ACVRL1) were transiently knocked-down using a single dose of siRNA. The function of the siRNA was confirmed in monolayer culture (Supplementary Figure 1).

**Figure 7:**
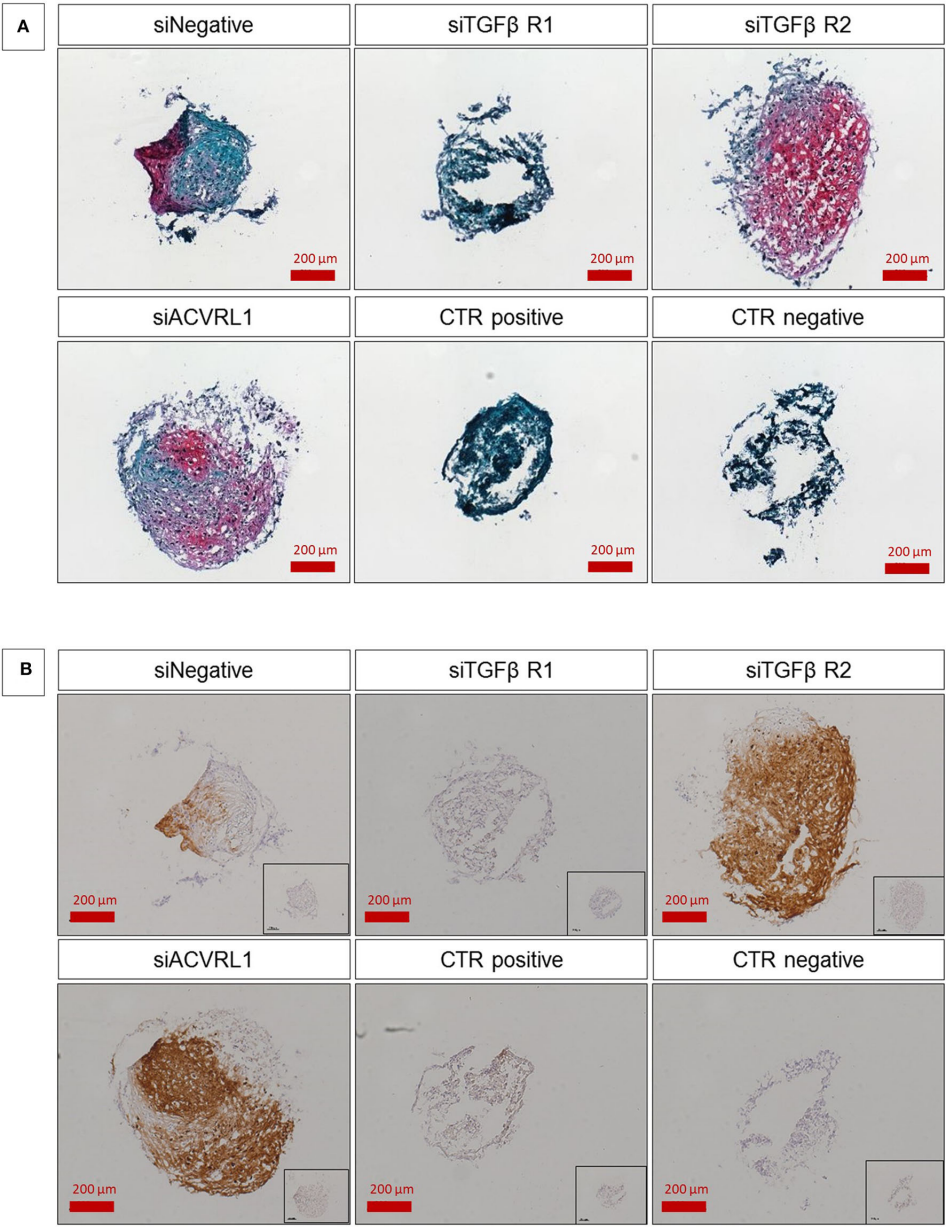
Safranin-O/fast green staining **(A)** and type II collagen/hematoxylin-mayer immunostaining **(B)** of hMSC pellets. SiNegative shows pellet that has been transfected with scramble control and was cultured with TGFβ; siTGFβR1, siTGFβR2, and siACVRL1 were transfected with the respective siRNA and was cultured with TGFβ; CTR positive has been not transfected but was cultured with TGFβ; CTR negative has been not transfected and was not cultured with TGFβ. The intensity of safranin-O (Red) staining is directly proportional to the proteoglycan content inside the pellet, while green structures represent the counterstaining with fast green solution **(A)**. Brown color represents the positive reaction to type II collagen, counter stained with Hematoxylin-Mayer; Inset images show the respective negative controls for the immunostaining **(B)**. The figures are representative of five separate experiments using five different donors. Scale bar for 10× objective = 200 μm.

For those donors that showed a high AUR, no changes during differentiation upon silencing of TGFβ-Rs was observed (data not shown). On the contrary, all donors (*n* = 5/5) with low initial AUR positively responded to the silencing of TGFβ-RII, with a marked enhancement of matrix deposition that was clearly observed by safranin-O staining and COL2A1 protein expression revealed by immunohistochemistry (Figure 7). This confirms our previous findings on TGFβ-Rs ratio and demonstrates that it is possible to interfere with the fate associated with the TGFβ-Rs profile by converting hMSCs into a pro-chondrogenic state. It also suggests that high expression of TGFβ-RII may be a reason for poor chondrogenic differentiation, at least in cells aged by *in vitro* culture.

Interestingly, while the silencing of TGFβ-RI did not significantly alter differentiation in comparison with the negative scramble control, ACVRL-I knockdown also led to increased chondrogenic potential (Figure 7). Treatment of functional MSC populations with siRNA did not have a noticeable effect (data not shown). We also investigated the osteogenic potential relative to the receptor ratio and no correlation was observed (Supplementary Figure 2).

## Discussion

Though widely used, CD markers do not offer insights into MSC function and this has led to the search for more functional outcome parameters. We had previously shown a change in Runx2/Sox9 ratio to be a reliable predictor of MSC osteogenesis (Loebel et al., 2015), however this still requires a osteogenic stimulus to be applied and a period of time to wait for changes to occur. Varas et al. have proposed alpha10 integrin as a marker of chondrogenic potency (Varas et al., 2007). More recently the group of Hollander proposed receptor tyrosine kinase-like orphan receptor 2 (ROR2) as a potency marker for human MSC chondrogenesis (Dickinson et al., 2017). TWIST1 has been proposed as a marker of MSC secretory phenotype that can be manipulated during monolayer expansion by exposure to fibroblast growth factor 2 or interferon gamma (Boregowda et al., 2016). Therefore, the expression of TWIST 1 has been proposed as a clinical indication prediction scale, with high twist leading to a more angiogenic phenotype, while low TWIST1 results in an immuno-modulatory phenotype (Boregowda et al., 2016). The downregulation of TWIST1 has also been demonstrated during chondrogenic differentiation (Cleary et al., 2017).

Real-time PCR is a precise method for determination of gene expression of a target gene. As a consequence, ΔΔCT determinations (Livak and Schmittgen, 2001; Schefe et al., 2006) have become a standard measurement, and are used in cell differentiation experiments to discern the differentiation state of a given cell population. Pooling results of analyses for cells from multiple donors (biological replicates) and within a given experimental setup (technical replicates) to provide overall means and standard deviations for comparison between groups is commonly used to detect a consistent result that should be repeatable. Unfortunately, this approach can hide patterns that are present in subgroups, masking explanations for donor to donor variation (Stoddart et al., 2012). Donor variation in primary human isolates of MSCs is widely accepted, yet the classical approach of pooling data and comparing the mean and standard deviation persists. Using such statistical analyses, we initially concluded that there were no changes in TGFβ receptor profiles that correlated with hMSC chondrogenic potential (Figure 1). However, when analyzing the data on a donor by donor basis, clear trends started to emerge and an increase of *SD* over time was an indicator that the marker under investigation could be of interest. Chondrogenic media typically contains a standardized concentration of TGFβ, yet the donor response varies. We hypothesized that the variation seen was due to changes in receptor expression, leading to changes in bioavailability. In our experiments we initially started comparing the changes in BMP and TGFβ receptors in MSCs during passaging using analyses of populations containing multiple donors by comparing means and *SD* of the whole cohort. Using this method, we could not detect significant differences in BMP-receptors except for a higher standard deviation in BMP-R1B and BMP-RII expression with passage (Figure 1). While investigating *in vivo* aging, (Moerman et al., 2004) found similar results with MSCs isolated from mice at different ages, with Moerman noting a decrease BMP-R1B. However, no direct link to function could be observed.

For TGFβ-receptors we could identify a slight increase in TGFβ-RII expression with a trend of TGFβ-RI decreasing, but with neither change being consistent, nor individually being correlated with differentiation (Figure 1). As the receptors signal as a hetero-tetrameric TGFβ-RI/ TGFβ-RII complex we analyzed the ratio on an individual donor level and noted that cell isolates with lower chondrogenicity expressed a lower TGFβ-RI/TGFβ-RII ratio compared with more chondrogenic cells, as assessed by histology and PCR (Figure 2). Moreover, as cells were *in vitro* aged using monolayer expansion the ratio changed over time in a donor dependent manner and a decrease correlated with a decrease in chondrogenic potential. Calculation of the $R=2^{-\left(\Delta Ct_{\text{hTGFβ}-\text{RI }}-\Delta Ct_{\text{hTGFβ}-\text{RII}}\right)}\;$ratio showed the same effects with the simplification that a value could be calculated at any passage without the need of a calibrator. It was shown that MSCs from aged hearts show a lower TGFβ-RI expression (Cieslik et al., 2014). Dexheimer et al. found that TGFβ-RII decreased in expression in MSCs during chondrogenic differentiation (Dexheimer et al., 2016), which suggests that a low TGFβ-RII might be better for chondrogenic differentiation and might be the reason why older cells tend to be less potent for differentiation than younger.

An AUR > 0.136 AUR (Arbitrary Unit Ratio) for the $2^{-\left(\Delta Ct_{\text{hTGFβ}-\text{RI }}-\Delta Ct_{\text{hTGFβ}-\text{RII}}\right)}$ ratio is a good baseline for cells with good chondrogenic differentiation potential (Figure 3). Only one cell population with a ratio of 0.123991 was incorrectly characterized as non-responsive, when in fact it was chondrogenic (Supplementary Table 3). As more donors are investigated the actual cut off value may be further refined, but the principle of a low ratio leading to poor chondrogenesis is now established. Whether other laboratories will obtain exactly the same ratio is still to be seen. Furthermore, we are currently investigating other MSC cell sources to determine how broadly applicable to chondrogenesis prediction will be to other cell types. Similarly to the ROR2 chondrogenic marker, this chondrogenic ratio did not correlate with osteogenic potential [(Dickinson et al., 2017) and Supplemental Figure 2], suggesting specific markers may need to be discovered for specific phenotypes.

The identification of a threshold above which chondrogenesis reliably occurs offers the potential to enhance chondrogenic potential of previously non-responsive human donors. A transient siRNA interference of TGFBR2 increased the ratio and led to improved chondrogenesis in all donors tested (Figure 7, Supplementary Table 3). This is an advantage for autologous therapies and would allow cells from all patients to be used with an increased chance of success. It would also offer a method to produce a more reproducible allogeneic cell source. The lack of any detrimental effect on siRNA knockdown on functional cell populations suggests that a receptor manipulation step could become a standardized part of any treatment protocol, thus allowing for a standardized approach. As ACVRL1 knockdown also leads to reversal of poor donor phenotype (Figure 7), it suggests the underlying mechanism is limiting the bioavailability of the TGFB/TGFBR2 complex to favor heterodimerization with TGFBR1. siRNA knockdown of TGFBRIII has also been shown to improve TGFβ3 induced chondrogenesis (Zheng et al., 2018). Of note, the siRNA induced knockdown of TGFBRIII was performed using a lentiviral expression system, whereas within this study a single dose applied using electroporation, was sufficient to direct the chondrogenic response over weeks. This would suggest that fate decisions can be taken within the first 24 h, and then become self-propagating, offering the opportunity to manipulate cell differentiation by early and transient modification of cell phenotype.

TGFBRII initially binds TGFβ protein and then forms a complex with an additional receptor. While TGF-β signaling is normally associated with recruitment of the ALK5 (TGFBR1) receptor, activating the SMAD 2/3 pathway, it is also known that TGFβ can signal via ALK1 (ACVRL1), leading to SMAD 1/5/8 activation (Blaney Davidson et al., 2009). Both receptors are present in MSCs [our data and (de Kroon et al., 2015)] and both receptors are needed for chondrogenesis (Hellingman et al., 2011). In other cell types it has been shown that TGF-β signaling is dose dependent with a low dose favoring SMAD 2/3 signals via ALK5, while increasing TGFβ concentration shifts the balance in favor of ALK1 and SMAD 1/5/8 signals (Remst et al., 2014). This has led us to the hypothesis that the relative level of TGFBRII vs. TGFBRI on the cell is defining when classical chondrogenic media cocktails containing 10 ng TGFβ are used. As TGFBRII binds TGFβ, it preferentially recruits TGFBR1 and activates the SMAD 2/3 pathway. If ligand bound TGFBRII is still available once the TGFBRI has been recruited, it is then able to recruit increasing numbers of ALK1 receptors, with increasing SMAD 1/5/8 signaling. By downregulating TGFBRII the balance is shifted in favor of the TGFBRI recruitment, and despite the excess of TGFβ there is no available TGFBRII to bind the ligand and complex with ALK1.

## Conclusion

In the era of personalized medicine, patient specific quantitative measures will be required that do not rely on statistical evaluation. Determination of the TGFβ-RI/TGFβ-RII ratio in hMSCs during expansion is a relatively easy method to screen MSCs for their chondrogenic differentiation properties. While MSCs at higher passages with lower differentiation potential show a lower quotient, the quotient is higher in cells with greater chondrogenicity. We defined a 2^−Δ*CtTGFβ*−*RI*^/2^−Δ*CtTGFβ*−*RII*^ ratio value of ~0.136 as the threshold for indicating an hMSC cell preparation has poor chondrogenicity. Once a potential mechanism was established, there was the opportunity to modify the receptor profile and assess the effect of chondrogenic differentiation. Transient inhibition of TGFBRII expression by siRNA treatment reliably enhanced the chondrogenic differentiation of all non-chondrogenic donors. Increasing the TGFβ-RI/TGFβ-RII ratio by temporary knockdown of TGFβ-RII via siRNA prior to differentiation can increase the differentiation potential of hMSCs. Donors with good differentiation quality are not affected by this knockdown. The ability to increase chondrogenicity of a given preparation will enhance the possibility to use a patient's own cells for autologous cell therapies involving cartilage tissue engineering.

## Data Availability Statement

All datasets generated for this study are included in the article/Supplementary Material.

## Ethics Statement

The studies involving human participants were reviewed and approved by ethics board of the University of Freiburg, permission number 135/14. All samples were harvested after signed informed consent.

## Author Contributions

RR, VB, and MS: conception and design, collection and assembly of data, data analysis, and interpretation. DK, FD, RS, and BJ: sample collection and interpretation, final approval of manuscript. RR, VB, BJ, and MS: writing and final original draft of manuscript. DK, FD, RS, BJ, and MA: review & editing of manuscript. All authors contributed to the article and approved the submitted version.

## Conflict of Interest

The AO Foundation has a patent pending on the TGF-receptor ratio and reversal of poor donor chondrogenesis. None of the authors will personally benefit should the patent application be successful.
