# Supplementary material for: Predicting and Promoting Human Bone Marrow MSC Chondrogenesis by Way of TGFβ Receptor Profiles: Toward Personalized Medicine
Source: Front Bioeng Biotechnol. 2020 Jun 26;8:618. doi: 10.3389/fbioe.2020.00618 (PMC7333220; doi:10.3389/fbioe.2020.00618)
Supplement: Supplementary file 1 [file Table_1.docx]

| Name of Gene | Primer order code or Sequence | Type | Producer |
| --- | --- | --- | --- |
| hACVRL1 | Hs01050825_s1 | On Demand | Applied Biosystem |
| hACVR1 | Hs00153836_m1 | On Demand | Applied Biosystem |
| hACVR1B | Hs00244715_m1 | On Demand | Applied Biosystem |
| hACVR1C | Hs00899854_m1 | On Demand | Applied Biosystem |
| hACVR2A | Hs00155658_m1 | On Demand | Applied Biosystem |
| hACVR2B | Hs00609603_m1 | On Demand | Applied Biosystem |
| hBMPR1A | Hs01034913_g1 | On Demand | Applied Biosystem |
| hBMPR1B | Hs01010965_m1 | On Demand | Applied Biosystem |
| hBMPR2 | Hs00176148_m1 | On Demand | Applied Biosystem |
| hTGFBR1 | Hs00610320_m1 | On Demand | Applied Biosystem |
| hTGFBR2 | Hs00234253_m1 | On Demand | Applied Biosystem |
| hSOX9 | Hs00165814_m1 | On Demand | Applied Biosystem |
| hACAN | Hs01050178_m1 | On Demand | Applied Biosystem |
| hRPLP0 fwd | 5'-TGG GCA AGA ACA CCA TGA TG-3' | Primer & Probe | MicroSynth |
| hRPLP0 rev | 5'-CGG ATA TGA GGC AGC AGT TTC-3' | Primer & Probe | MicroSynth |
| hRPLP0 Pr | 5'-AGG GCA CCT GGA AAA CAA CCC AGC-3' | Primer & Probe | MicroSynth |
| hCOL2A1 fwd | 5'-GGC AAT AGC AGG TTC ACG TAC A-3' | Primer & Probe | MicroSynth |
| hCOL2A1 rev | 5'-GAT AAC AGT CTT GCC CCA CTT ACC-3' | Primer & Probe | MicroSynth |
| hCOL2A1 Pr | 5'-CCT GAA GGA TGG CTG CAC GAA ACA TAC-3' | Primer & Probe | MicroSynth |
| hCOL10A1 fwd | 5'-ACG CTG AAC GAT ACC AAA TG-3' | Primer & Probe | MicroSynth |
| hCOL10A1 rev | 5'-TGC TAT ACC TTT ACT CTT TAT GGT GTA-3' | Primer & Probe | MicroSynth |
| hCOL10A1 Pr | 5'-ACT ACC CAA CAC CAA GAC ACA GTT CTT CAT TCC-3' | Primer & Probe | MicroSynth |
| hMMP13 fwd | 5'-CGG CCA CTC CTT AGG TCT TG-3' | Primer & Probe | MicroSynth |
| hMMP13 rev | 5'-TTT TGC CGG TGT AGG TGT AGA TAG-3' | Primer & Probe | MicroSynth |
| hMMP13 Pr | 5'-CTC CAA GGA CCC TGG AGC ACT CAT GT-3' | Primer & Probe | MicroSynth |

Supplemental table 1
