# Supplementary material for: Predicting and Promoting Human Bone Marrow MSC Chondrogenesis by Way of TGFβ Receptor Profiles: Toward Personalized Medicine
Source: Front Bioeng Biotechnol. 2020 Jun 26;8:618. doi: 10.3389/fbioe.2020.00618 (PMC7333220; doi:10.3389/fbioe.2020.00618)
Supplement: Supplementary file 2 [file Table_2.docx]

| **Uniformity of Saf-O** | **Assessment** | **Score** |
| --- | --- | --- |
|  | Poor | 0 |
|  | Low | 1 |
|  | moderate | 2 |
|  | High | 3 |
| **Darkness of Saf-O** |  |  |
|  |  |  |
|  | Poor | 0 |
|  | Low | 1 |
|  | moderate | 2 |
|  | High | 3 |
| **Density of cells** |  |  |
|  | Poor | 0 |
|  | Low | 1 |
|  | moderate | 2 |
|  | High | 3 |
| **Shape of pellet** |  |  |
|  | Round | 0 |
|  | not round | 1 |

Supplementary table 2. Histological score description.
