# Supplementary material for: Predicting and Promoting Human Bone Marrow MSC Chondrogenesis by Way of TGFβ Receptor Profiles: Toward Personalized Medicine
Source: Front Bioeng Biotechnol. 2020 Jun 26;8:618. doi: 10.3389/fbioe.2020.00618 (PMC7333220; doi:10.3389/fbioe.2020.00618)
Supplement: Supplementary file 3 [file Table_3.docx]

| Donor | Year of Birth | Passage | Ratio | Chondrogenic potential | Histological score | SiRNA inverted | Reverted situation |
| --- | --- | --- | --- | --- | --- | --- | --- |
|  |  |  |  |  |  |  |  |
| 147 | 1970 | p2 | 0.061805 | no | 2 | N/P |  |
| 147 |  | p4 | 0.091174 | no | 2 | yes | yes |
|  |  |  |  |  |  |  |  |
| 143 | 1962 | p2 | 0.123991 | yes | 6 | N/P |  |
| 143 |  | p4 | 0.149042 | yes | 4 | N/P |  |
| 143 |  | p6 | 0.07661 | no | 1 | yes | yes |
|  |  |  |  |  |  |  |  |
| 120 | 1939 | p2 | 0.168311 | yes | 6 | N/P |  |
| 120 |  | p4 | 0.229862 | yes | 7 | N/P |  |
| 120 |  | p6 | 0.285203 | yes | 8 | N/P |  |
| 120 |  | p8 | 0.072031 | no | 1 | N/P |  |
|  |  |  |  |  |  |  |  |
| 168 | 1952 | p2 | 0.027427 | no | 1 | yes | yes |
| 168 |  | p4 | 0.013382 | no | 1 | N/P |  |
| 168 |  | p6 | 0.020142 | no | 1 | N/P |  |
|  |  |  |  |  |  |  |  |
| 170 | 1958 | p2 | 0.282732 | yes | 8 | yes | no |
| 170 |  | p4 | 0.40052 | yes | 10 | N/P |  |
| 170 |  | p6 | 1.199033 | yes | 8 | N/P |  |
| 170 |  | p8 | 0.113863 | no | 5 | N/P |  |
| 170 |  | p10 | 0.060778 | no | 2 | N/P |  |
|  |  |  |  |  |  |  |  |
| 195 | 1939 | p2 | 0.166582 | yes | 7 | N/P |  |
| 195 |  | p4 | 0.024063 | no | 1 | N/P |  |
| 195 |  | p6 | 0.029769 | no | 1 | N/P |  |
| 195 |  | p8 | 0.050305 | no | 1 | N/P |  |
|  |  |  |  |  |  |  |  |
| 200 | 1967 | p2 | 0.234267 | yes | 8 | N/P |  |
|  |  |  |  |  |  |  |  |
| 92 | 1940 | p3 | 0.060776 | no | 2 | yes | yes |
|  |  |  |  |  |  |  |  |
| 132 | 1966 | p4 | 0.08 | no | 1 | yes | yes |

Supplementary Table 3. All siRNA experiments are described. N/P = Not performed.
