# Supplementary material for: Predicting and Promoting Human Bone Marrow MSC Chondrogenesis by Way of TGFβ Receptor Profiles: Toward Personalized Medicine
Source: Front Bioeng Biotechnol. 2020 Jun 26;8:618. doi: 10.3389/fbioe.2020.00618 (PMC7333220; doi:10.3389/fbioe.2020.00618)
Supplement: Supplementary file 4 [file Image_1.pdf]

Supplementary Figure 1

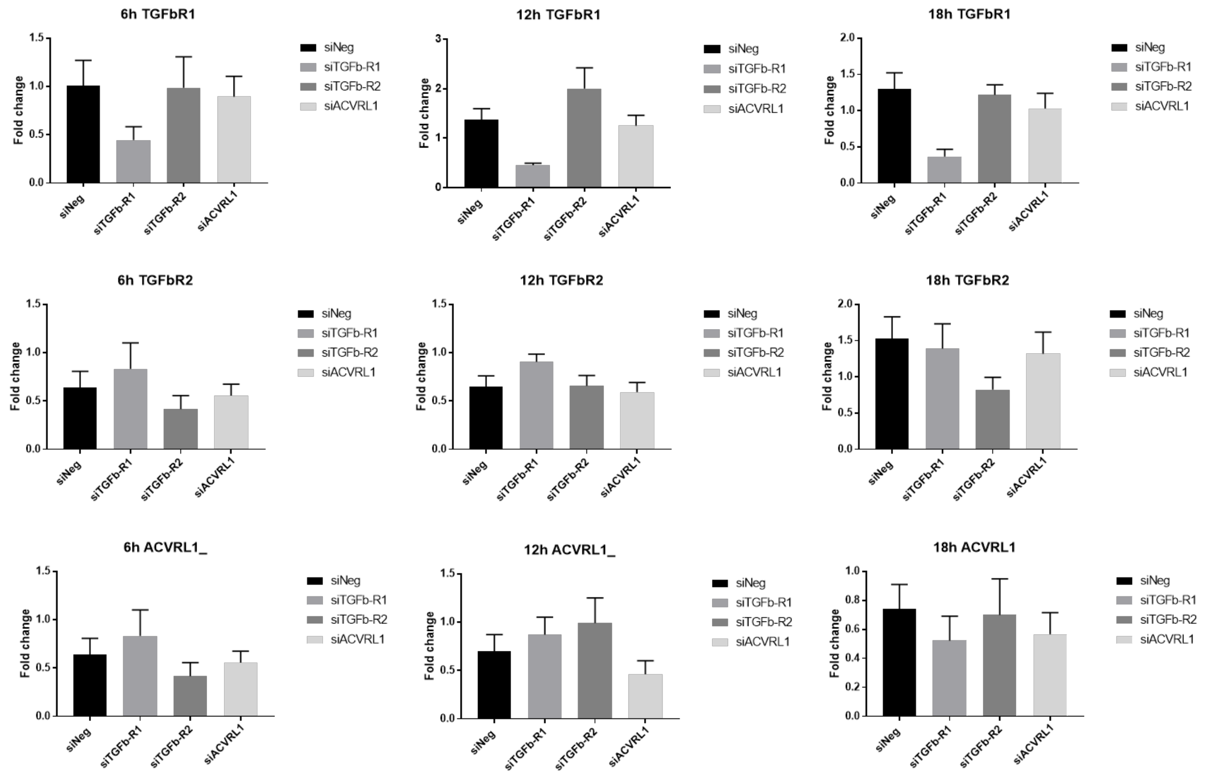

Supplementary Figure 1. Modulation of TGFB receptors knocking-down by silencing-RNA after 6-12 and 18 hours after transfection*. Receptors were transiently knocked-down using the respective siRNA. The amounts of TGFBR1(A), TGFBR2 (B), ACVRL1 (C) mRNA after 6-12 and 18 transfection was normalized to ribosomal protein large, P0 (RPLP0). The mRNA expression of receptors over time were plotted as* $2^{(-\Delta\Delta Ct)}$ *(mean ± SD; n = 5) as a fold change to the T0 before transfection.*
