# Supplementary material for: Predicting and Promoting Human Bone Marrow MSC Chondrogenesis by Way of TGFβ Receptor Profiles: Toward Personalized Medicine
Source: Front Bioeng Biotechnol. 2020 Jun 26;8:618. doi: 10.3389/fbioe.2020.00618 (PMC7333220; doi:10.3389/fbioe.2020.00618)
Supplement: Supplementary file 5 [file Image_2.pdf]

Supplementary Figure 2

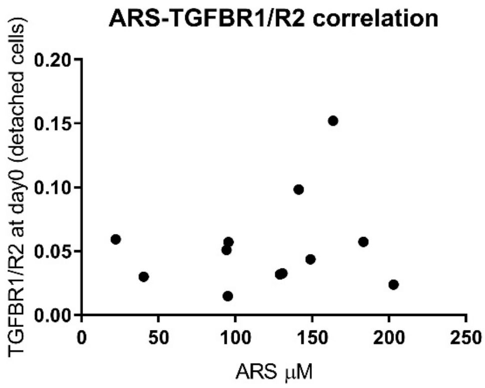

Supplementary Figure 2. Correlation of TGFBR1/TGFBR2 ratio to alizarin red quantification after 21 days of osteogenic differentiation. *Four donors at various passages (n=12) were induced towards osteogenic differentiation for 21 days and the monolayer stained with alizarin red for calcification. The bound dye was dissolved and the released colour measured spectrophotometrically. No correlation between alizarin red staining and the receptor ratio was detected.*
